# Synthesis and Antioxidant Activity of Cationic 1,2,3-Triazole Functionalized Starch Derivatives

**DOI:** 10.3390/polym12010112

**Published:** 2020-01-05

**Authors:** Yuan Chen, Xiguang Liu, Xueqi Sun, Jingjing Zhang, Yingqi Mi, Qing Li, Zhanyong Guo

**Affiliations:** 1 School of Chemistry and Materials Science, Ludong University, Yantai 264025, Chinaxgliu1986@163.com (X.L.); 2 Key Laboratory of Coastal Biology and Bioresource Utilization, Yantai Institute of Coastal Zone Research, Chinese Academy of Sciences, Yantai 264003, China; 3 University of Chinese Academy of Sciences, Beijing 100049, China

**Keywords:** starch, chemical modification, 1,2,3-triazole, antioxidant activity

## Abstract

In this study, starch was chemically modified to improve its antioxidant activity. Five novel cationic 1,2,3-triazole functionalized starch derivatives were synthesized by using “click” reaction and *N*-alkylation. A convenient method for pre-azidation of starch was developed. The structures of the derivatives were analyzed using FTIR and ^1^H NMR. The radicals scavenging abilities of the derivatives against hydroxyl radicals, DPPH radicals, and superoxide radicals were tested in vitro in order to evaluate their antioxidant activity. Results revealed that all the cationic starch derivatives (2a–2e), as well as the precursor starch derivatives (1a–1e), had significantly improved antioxidant activity compared to native starch. In particular, the scavenging ability of the derivatives against superoxide radicals was extremely strong. The improved antioxidant activity benefited from the enhanced solubility and the added positive charges. The biocompatibility of the cationic derivatives was confirmed by the low hemolytic rate (<2%). The obtained derivatives in this study have great potential as antioxidant materials that can be applied in the fields of food and biomedicine.

## 1. Introduction

Natural polysaccharides with advantages of renewability, biocompatibility, and biodegradability are considered as potential substitutes for traditional fossil resources, which have been used to prepare a variety of functional materials, covering different fields such as biomedicine [1], pharmaceutical [2], food [3], textile [4], environmental protection [5], among others. Starch is one example of polysaccharides that are widely used.

Starch is the main dietary source of carbohydrates for human beings and the most abundant plant-derived storage polysaccharide. As a kind of natural polymer, starch is formed by condensation of glucose units through α-d-(1-4) and/or α-d-(1-6) glycoside bonds [6]. The design and synthesis of starch-based materials have been a hot topic due to their unique biocompatibility, biodegradability, and film-forming properties [7]. Drug delivery nanomaterials [8], implantable biomaterials [9], and active packaging materials [10] are the promising applications of starch-based materials.

Recently, more attention has been focused on free radicals and antioxidants. It is generally accepted that oxidative stress is closely related to many diseases, such as chronic prostatitis, chronic pelvic pain syndrome, male infertility, cardiovascular diseases, Alzheimer’s disease, and cancer [11]. Oxidative stress and free radicals are even part of a dominant theory in terms of the cause of aging [12]. Recently, it has been proved that oxidative stress is an essential factor in tissue response to biomaterials, often limiting their long-term biocompatibility and functioning [13]. Actually, several well-known and often-used biomaterials have been implicated in inducing local oxidative stress [13]. For this reason, biomaterials with intrinsical antioxidant activity have the advantages of avoiding the prolonged inflammatory response caused by oxidative stress, leading to a growing field of research. Besides, antioxidant packaging materials are useful for extending the shelf-life of products and improving food safety or sensory properties [14]. As a result, the attempt to improve the antioxidant activity of starch is significant in terms of the development of antioxidant materials applied in the fields of food and biomaterials.

The anhydroglucose unit (AGU) of starch provides three active hydroxyl groups, including one primary and two secondary hydroxyl groups, making it convenient for chemical modification of starch. Many efforts oriented towards different application purposes have been focused on the chemical modification of starch. Chemical modifications of starch mainly involve acid hydrolysis, cross-linking, acetylation/esterification, dual modification, oxidation, and grafting [15]. Starch and its chemically modified derivatives have been widely utilized in many areas, such as the edible films [16], paper and textile [17], biomedical [18], pharmaceutical [19], and chemical production [20] fields, as well as in the food industry. Previously, we tried to modify starch through the introduction of different active groups and obtained some derivatives with favorable bioactivity [21,22,23].

The concept of “click” reaction was first introduced by Sharpless et al. in 2001 [23]. Cu(I) catalyzed 1,3-dipolar azide–alkyne cycloaddition (CuAAC) is the most typical model of the “click” reaction, with advantages of mild reactive conditions, wide selection of solvents, highly efficient, and easy product separation [23]. It is broadly utilized in the synthesis of structurally and functionally diverse molecules, particularly for pharmaceuticals and advanced materials applications [23]. The practicality and reliability of this reaction make it more and more popular in polysaccharide derivatization. Plenty of polysaccharide derivatives have been synthesized via the “click” reaction, accordingly introducing different types of functional groups to polysaccharide [23,24]. In addition, the “click” reaction serves as a bridge to accomplish the copolymerization of two or more molecules, bringing about various types of advanced materials [25]. To carry out the “click” reaction, pre-azidation of polysaccharide is commonly prepared in the first step. NBS (*N*-bromosuccinimide) and PPh_3_ (triphenylphosphine) are generally used to activate the primary hydroxyl in polysaccharide [26]. However, the reactive conditions are severe (high temperature, avoiding light, inert gas protection), and the added activating reagents are many times (3–10 times) more abundant than polysaccharide [27]. Moreover, the excess NBS and Ph_3_P, together with the by-products of the reaction, are difficult to completely remove during products separation [28]. Previously, we reported the chloroacetylation of chitosan, starch, and inulin as important intermediates for further nucleophilic reaction [21,29,30]. The chloroacetylation was carried out in water and required a small number of regents (2 times more than polysaccharide). In addition, the reaction had other advantages such as mild reaction conditions (room temperature), convenient separation and purification of products, and a high yield and substitution degree. On this basis, sodium azide was designed as a nucleophile to attack the carbon (ClCH_2_-) according to SN2 reaction. Afterwards, azide starch reacted with some terminal alkynes through a “click” reaction, thus introducing 1,2,3-triazole to starch. Finally, *N*-alkylation of the triazole group was carried out, making starch positively charged.

This study mainly aims to improve the antioxidant activity of starch, thus providing theoretical support for the development of starch-based antioxidant materials utilized in the food and biomedical fields. For this purpose, a convenient method for pre-azidation of starch was developed. A total of five novel cationic 1,2,3-triazole functionalized starch derivatives were synthesized by using a “click” reaction and *N*-alkylation. The structures of the obtained derivatives were analyzed using FTIR and ^1^H NMR. The antioxidant activities of the synthesized derivatives were evaluated by testing their radicals scavenging activities against hydroxyl radicals, DPPH radicals, and superoxide radicals. Some explanations about the structure and activity relationship were presented. The biocompatibility of the cationic starch derivatives was confirmed by the low hemolytic rate (<2%).

## 2. Experimental

### 2.1. Materials

Potato starch with a weight-average molecular weight of 9.8 × 10^4^ Da, was purchased from Sinopharm Chemical Reagent Co., Ltd. (Shanghai, China). *N*-bromosuccinimide (NBS), triphenylphosphine (PPh_3_), and terminal alkynes including 2-propyn-1-ol, 3-butyn-1-ol, 3-butyn-2-ol, 4-pentyn-1-ol, and 5-hexyn-3-ol, were all purchased from the Sigma-Aldrich Chemical Corp (Shanghai, China). Ethanol, chloroacetyl chloride, triethylamine, dimethylsulfoxide (DMSO), and *N*,*N*-dimethyl formamide (DMF) were all purchased from Sinopharm Chemical Reagent Co., Ltd. (Shanghai, China). The reagents were of an analytical grade and were used without further purification.

### 2.2. Analytical Methods

Fourier Transform Infrared (FTIR) spectra of the samples were performed on a Jasco-4100 (Tokyo, Japan, provided by JASCO China (Shanghai) Co. Ltd., Shanghai, China). The wave number was recorded in the range of 4000–400 cm^−1^, using transmittance mode at a resolution of 4.0 cm^−1^.

^1^H NMR spectra of the samples were performed on a Bruker AVIII-500 Spectrometer (500 MHz, Switzerland, provided by Bruker Tech, and Serv. Co., Ltd., Beijing, China) at 25 °C taking DMSO-d6 as a solvent. The degrees of the substitution of the starch derivatives were determined based on the integration of the ^1^H NMR spectrum [31].

The ultraviolet-visible (UV-vis) spectra were recorded on a UV-vis spectrometer (TU-1810, provided by Puxi General Instrument Co., Ltd., Beijing, China).

### 2.3. Synthesis of Starch Derivatives

#### 2.3.1. Synthesis of Chloroacetyl Starch (CASC)

**Scheme 1:**
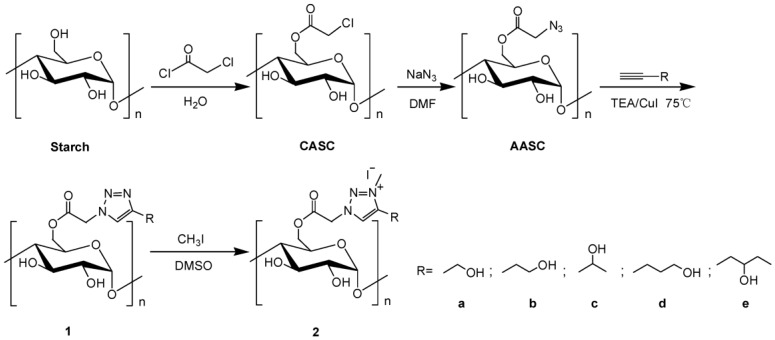
Synthesis of the starch derivatives.

CASC was synthesized according to Tan’s methods [23]. Starch (1.62 g, 10 mmol AGU) was firstly dispersed in 100 mL of deionized water at a 250 mL flask. Then, chloroacetyl chloride (20 mmol) was dropwise added. After stirring at room temperature for 12 h, the solution was evaporated to remove most of the water by a vacuum-rotary evaporation procedure. The crude product was precipitated in ethanol, then filtered and washed with ethanol three times. CASC was obtained after freeze drying under vacuum at −53 °C (Scheme 1).

#### 2.3.2. Synthesis of Azide Acetyl Starch (AASC)

A mixture of CASC (10 mmol) and sodium azide (30 mmol) were dissolved in 100 mL of DMF. The reaction was carried out at 80 °C for 4 h. Afterwards, the crude product was precipitated in excess ethanol. The solid was filtered and washed with ethanol then dried at −53 °C under vacuum to obtain azide acetyl starch (AASC) (Scheme 1).

#### 2.3.3. Synthesis of 1,2,3-Triazole Functionalized Starch Derivatives (1a–1e)

A mixture of AASC (1 mmol AGU), TEA (0.14 mL, 1 mmol), cuprous iodide (19 mg, 0.1 mmol), and terminal alkyne (3 mmol) dissolved in 25 mL of DMSO was stirred at 75 °C under argon atmosphere for 24 h to complete the reaction. The crude product was precipitated in excess ethanol and further purified in a Soxhlet apparatus with acetone for 48 h. The precursor starch derivatives (1a–1e) were obtained by freeze drying at −53 °C in vacuum (Scheme 1).

#### 2.3.4. Synthesis of 1,2,3-Triazolium Functionalized Starch Derivatives (2a–2e)

Above 1,2,3-triazole functionalized starch derivative (1a–1e, 1 mmol) was dissolved in 20 mL of DMSO. The solution was added dropwise with iodomethane (5 mmol), then warmed to 60 °C and vigorously stirred at reflux for 24 h. Afterwards, the solution was poured into excess acetone to isolate the product. After the solid was filtered and washed with ethanol and acetone three times, it was dialyzed against deionized water for 2 days to purify the product. The cationic 1,2,3-triazole functionalized starch derivatives (2a–2e) were obtained by freeze drying at −53 °C in vacuum (Scheme 1). 

### 2.4. Water Solubility

The test of water solubility was performed by the previous methods [23]. A total of 2 g starch or starch derivatives was stirred in 2 mL of distilled water at 25 °C until the dissolution equilibrium reached. Then, the solid was filtered, subsequently washed with ethanol, and freeze dried at −50 °C under a vacuum. The test was repeated three times and the values were expressed as mean ± the standard deviation (*SD*, *n* = 3). The water solubility (*S*) of starch and its derivatives were calculated by the following equation:

S = (2000 − m_1_)/2

where m_1_ means the weight of the undissolved parts (mg).

### 2.5. Antioxidant Assay

In general, antioxidant research is divided into in vivo antioxidant activity and in vitro antioxidant activity. Antioxidant research in vivo is based on antioxidant activity in vitro [32]. There are various spectrophotometric methods to assess the antioxidant activity in vitro, including hydroxyl radical scavenging assay, DPPH radical scavenging assay, and superoxide radical scavenging assay. These methods have been widely applied to assess the antioxidant activity of polysaccharide, along with its derivatives. In this article, antioxidant activities of the derivatives were evaluated according to the above-mentioned methods. Three kinds of methods were involved in order to comprehensively evaluate the antioxidant activity of the derivatives. The test samples included starch, 1a–1e, and 2a–2e. All used reagents were freshly prepared and the reaction was performed while avoiding light.

#### 2.5.1. Hydroxyl Radical Scavenging Ability

Hydroxyl radical scavenging assay was based on the Fenton oxidization [32]. Briefly, each sample (1 mL, 0.45 mg mL^−1^, 0.9 mg mL^−1^, 1.8 mg mL^−1^, 3,6 mg mL^−1^, and 7.2 mg mL^−1^) mixed with EDTA-Fe^2+^ (0.5 mL, 200 µM), potassium phosphate buffer (1 mL, 150 mM, pH 7.4), safranine T (1 mL, 0.23 µM), and H_2_O_2_ (1 mL, 60 µM), reacted at 37 °C for 30 min. The UV absorption of the mixture at 520 nm was measured subsequently. Three replications for each sample were performed and the hydroxyl radical scavenging ability was calculated using the following equation:

$$\mathrm{Scavenging}\;\mathrm{rate}\;(\%)=\frac{A_{\mathrm{sample}\;520\;\mathrm{nm}}-A_{\mathrm{blank}\;520\;\mathrm{nm}}}{A_{\mathrm{control}\;520\;\mathrm{nm}}-A_{\mathrm{blank}\;520\;\mathrm{nm}}}\times 100$$

where *A*_sample 520 nm_ was the absorbance of the sample, *A*_blank 520 nm_ was the absorbance of the blank group (distilled water replaced samples), and *A*_control 520 nm_ was the absorbance of the control group (distilled water replaced H_2_O_2_).

#### 2.5.2. DPPH Radical Scavenging Ability

The study of the DPPH radical scavenging ability was conducted using the methods developed by Tan [33]. In brief, 1 mL of each sample at different concentrations (0.3 mg mL^−1^, 0.6 mg mL^−1^, 1.2 mg mL^−1^, 2.4 mg mL^−1^, and 4.8 mg mL^−1^) mixed with 2 mL of DPPH solution (180 µM, dissolved in ethanol), was incubated at 25 °C for 30 min. Subsequently, the UV absorption of the mixture at 520 nm was recorded. Three replications for each sample were performed and the DPPH radical scavenging ability was calculated by the following equation:

$$\mathrm{Scavenging}\;\mathrm{rate}\;(\%)=(1-\frac{A_{\mathrm{sample}\;517\;\mathrm{nm}}-A_{\mathrm{control}\;517\;\mathrm{nm}}}{A_{\mathrm{blank}\;517\;\mathrm{nm}}})\times 100$$

where *A*_sample 517 nm_ was the absorbance of the sample, *A*_control 517 nm_ was the absorbance of the control (ethanol replaced DPPH solution), and *A*_blank 517 nm_ was the absorbance of the blank (distilled water replaced the samples).

#### 2.5.3. Superoxide Radical Scavenging Ability

The superoxide radical scavenging assay was performed following the methods developed by Tan [33]. Each sample (1.5 mL, 0.3 mg mL^−1^, 0.6 mg mL^−1^, 1.2 mg mL^−1^, 2.4 mg mL^−1^, and 4.8 mg mL^−1^) was seriatim added with NADH (0.5 mL, 456 μM, in Tris–HCl buffer with PH of 8.0), NBT (0.5 mL, 300 μM, in Tris–HCl buffer with PH of 8.0), and PMS (0.5 mL, 60 μM, in Tris–HCl buffer with PH of 8.0). The mixed solution was kept in dark at room temperature for 5 min. The UV absorption was measured at 560 nm. The test was repeated for three times at the same conditions. The superoxide radical scavenging ability was calculated using the following equation:

$$\mathrm{Scavenging}\;\mathrm{rate}\;(\%)=(1-\frac{A_{\mathrm{sample}\;560\;\mathrm{nm}}-A_{\mathrm{control}\;560\;\mathrm{nm}}}{A_{\mathrm{blank}\;560\;\mathrm{nm}}})\times 100$$

where *A*_sample 560 nm_ was the absorbance of the sample, *A*_control 560 nm_ was the absorbance of the control (distilled water replaced NADH) and *A*_blank 560 nm_ was the absorbance of the blank (distilled water replaced the samples).

### 2.6. Hemolysis Assay

The hemolysis assay was performed following reference [34] with appropriate modification. Briefly, fresh whole blood from healthy rabbit was collected in heparinized-tubes and centrifuged at 3000 rpm for 4 min. The plasma was separated and the erythrocyte was washed repeatedly with saline solution until the supernatant was colorless. The test samples including native starch, 1a–1e, and 2a–2e, were dissolved in saline solution. Afterwards, 0.2 mL of erythrocyte was added to the solution to make the final test concentrations of the samples at 1000, 500, 100, 50, 10, and 1 μg/mL. All the tested tubes were incubated at 37 °C for 1 h. Then, the suspension was centrifuged at 1000 rpm for 10 min and absorbance of the supernatant of each tube was measured at 540 nm. Triton X-100 and saline solution were used as positive (CP, 100% lysis) and negative (CN, 0% lysis) controls, respectively. The test was repeated three times. Hemolysis rate was calculated using the following equation:

$$\mathrm{Hemolysis}\;(\%)=\frac{\mathrm{Sample}\;\mathrm{absorbance}-\mathrm{CN}\;\mathrm{absorbance}}{\mathrm{CP}\;\mathrm{absorbance}-\mathrm{CN}\;\mathrm{absorbance}}\times 100$$

### 2.7. Statistical Analysis

The data of the antioxidant activities were processed by Excel, Origin, and SPSS to provide the results. The data were analyzed using an analysis of variance. When *p* < 0.05, the results were considered statistically significant.

## 3. Results and Discussion

### 3.1. Structure Characterization

**Figure 1:**
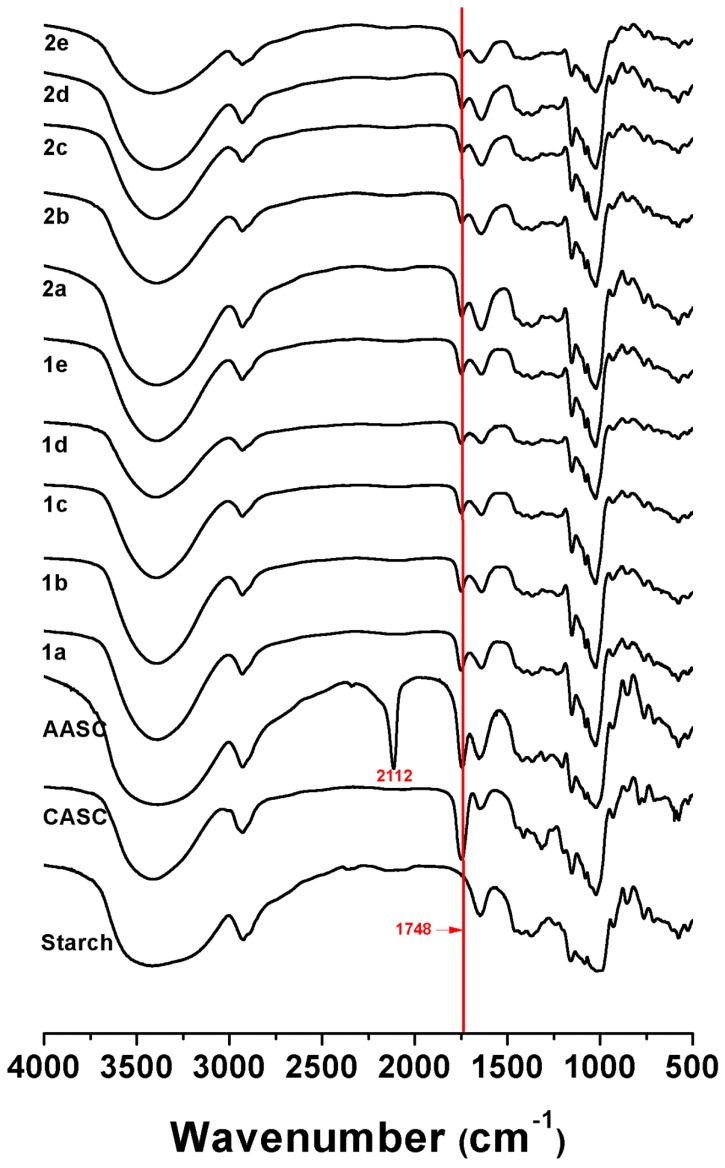
Fourier Transform Infrared (FTIR) spectra of starch, CASC, AASC, 1a–1e, and 2a–2e.

**Figure 2:**
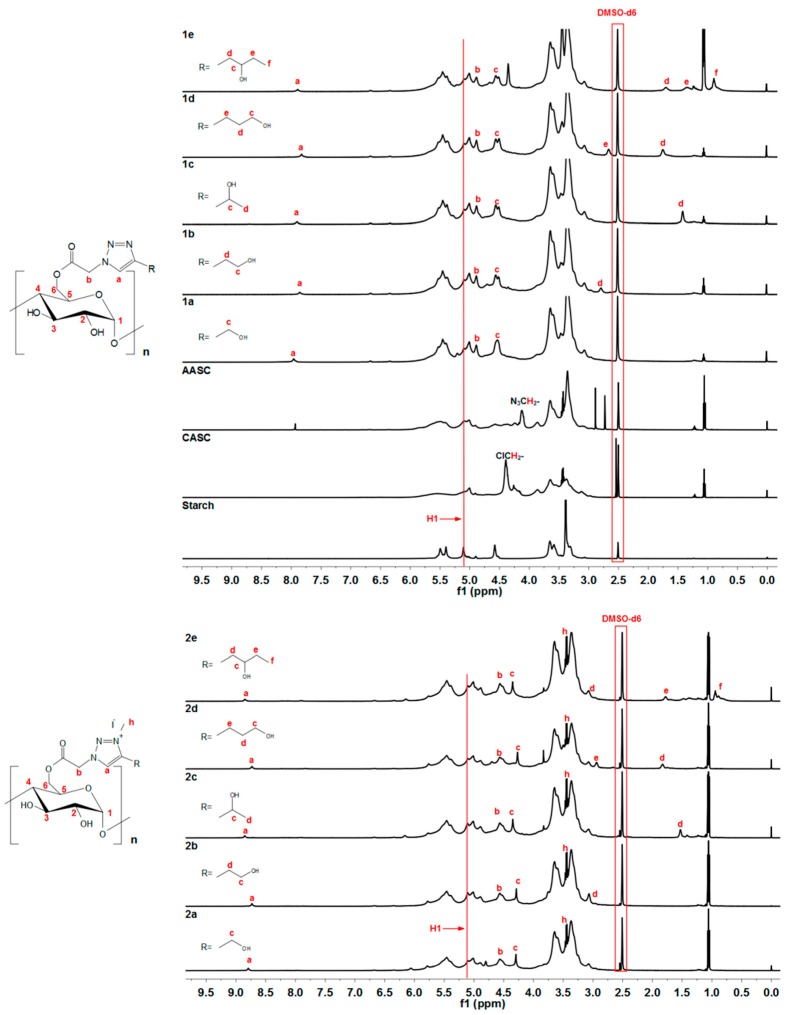
^1^H NMR spectra of starch, CASC, AASC, 1a–1e, and 2a–2e.

The structure of starch and its derivatives were characterized by using FTIR (Figure 1) and ^1^H NMR spectra (Figure 2).

Figure 1 showed the FTIR spectra of starch, CASC, AASC, 1a–1e, and 2a–2e. Characteristic peaks of pure starch included *ν*(O–H) 3428 cm^−1^, *ν*(C–H) 2927 cm^−1^, *δ*(O–H) 1427 cm^−1^, *δ*(C–H) 1373 cm^−1^, and an absorption band *ν*(C–O) and *ν*(C–C) 1200–990 cm^−1^. After the chloride acetylation, a new peak at 1748 cm^−1^ was assigned to *ν*(–COO–) of the chloroacetyl group. Regarding AASC, a strong peak at 2112 cm^−1^ was assigned to the azide group. For 1a–1c, the peak at 2112 cm^−1^ disappeared completely, because the azide group in AASC was transformed to 1,2,3-triazole, which indicated the “click” reaction was successfully carried out.

^1^H NMR spectra in Figure 2 was applied to further confirm the structure of the derivatives. According to literature [34], the signals of starch backbone were located at 3.0–5.7 ppm. The signal of α-anomeric hydrogen (H1) of the native starch was at 5.1 ppm and six other AGU protons (H2–H6) were around 3.2–4.0 ppm [35]. After the chloride acetylation of starch, a new signal was observed at 4.4 ppm in the ^1^H NMR spectra of CASC, which was contributed to the hydrogen of –CH_2_Cl. In the ^1^H NMR spectra of AASC, the new signal at 4.1 ppm belonged to the hydrogen of –CH_2_N_3_, indicating that the azide group had been successfully grafted onto starch. In the ^1^H NMR spectra of 1a–1c, the hydrogen at C5 position in the triazole ring showed absorption at 7.8–8.0 ppm, indicating the successful introduction of 1,2,3-triazole group to starch. In addition, the hydrogen of methylene between triazole and ester group showed absorptions at around 4.9 ppm. Other methylenes also showed signals at 4.5 ppm (in 1a), 2.8 ppm and 4.5 ppm (in 1b), 1.4 ppm and 4.5 ppm (in 1c), 1.7 ppm, 2.6 ppm, and 4.5 ppm (in 1d), and finally 0.9 ppm, 1.3 ppm, 1.7 ppm, and 4.5 ppm (in 1e). After alkylation with methyl iodide, some changes of the chemical shifts could be observed from the figure. For example, the signal of the hydrogen at the C5 position in the triazole ring disappeared at 7.8–8.0 ppm, and instead appeared at 8.7–8.9 ppm. A new signal at around 3.4 ppm in 2a–2e contributed to the hydrogen of methyl in the cationic 1,2,3-triazole group. In addition, all the signals of methylene mentioned above were moved compared to those signals in 1a–1e.

### 3.2. Degree of Substitution (DS)

In this study, the DS of starch derivatives (2a–2e) was calculated by using the ^1^H NMR spectrum. This was based on the position and intensity of the signal of the α-anomeric hydrogens (H1) at 5.1 ppm [31]. The results are summarized in Table 1. From the table, the DS of CASC was above 1.0, indicating that the chloride acetylation was also performed at the secondary hydroxyl groups of starch apart from the C6-OH, as the chloroacetyl chloride was an extremely active reagent. The DS of all the starch derivatives was located at 0.2–0.28. The relatively low DS was probably due to the hydrolysis of ester groups. Through the comparation of the DS between the cationic starch derivatives (2a–2e) and the precursor starch derivatives (1a–1e), it was found that the alkylation was quite thorough.

### 3.3. Water Solubility

The water solubilities of starch and its derivatives, as well as the intermediates in distilled water at 25 °C were summarized in Table 1. Soluble starch was insoluble in neutral water at room temperature. After chloride acetylation and azidation, the water solubility of CASC and AASC was improved. All the starch derivatives exhibited favorable water solubility at room temperature. The solubilities of all the precursor starch derivatives (1a–1e) in water were above 480 mg/mL. After alkylation, the water solubilities of all the cationic starch derivatives (2a–2e) were improved obviously, exceeding 800 mg/mL. The reason was discussed as follows. After chemical modification of starch, the effect of intramolecular and intermolecular hydrogen bonding was greatly weakened. Additionally, the enhanced water solubility also benefited from the addition of hydrophilic triazole group. After alkylation, cationic ammonium salts further improved the water solubility of starch derivatives.

### 3.4. Antioxidant Activity

**Figure 3:**
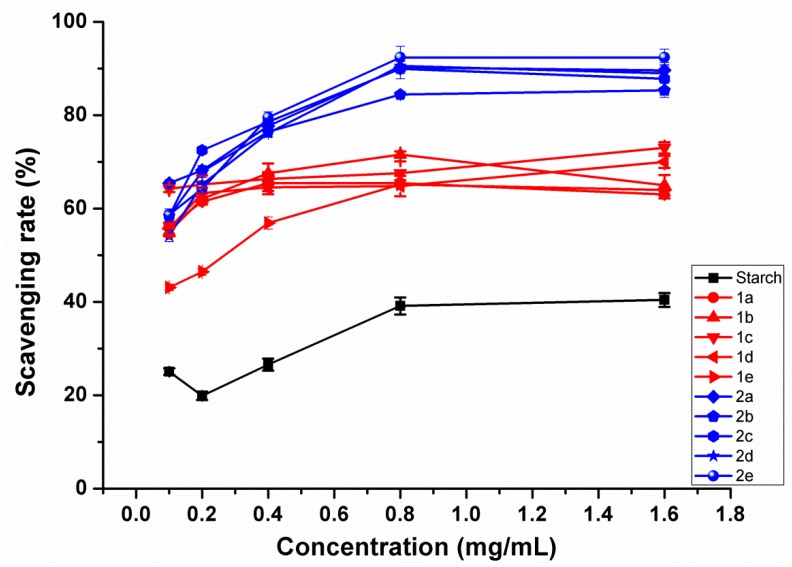
Hydroxyl radical scavenging activity of starch and starch derivatives.

It is generally recognized that the hydroxyl radical is generated from H_2_O_2_ by the Fenton reaction [36]. Hydroxyl radical is the most reactive product of ROS formed by successive 1-electron reductions of molecular oxygen (O_2_) in cell metabolism [36]. It is an extremely strong oxidative free radical, which could lead to cell death or mutation through the destruction of purine and pyrimidine in deoxyribonucleic acid (DNA) [36], and is primarily responsible for the cytotoxic effects observed in aerobic organisms extending from bacteria to plants and animals [36]. Figure 3 showed the hydroxyl radical scavenging ability of starch and its derivatives. As seen in the figure, all the starch derivatives exhibited an enhanced scavenging ability compared to native starch. The scavenging rates of the derivatives against hydroxyl radicals were enhanced with the increase of their concentrations at 0.1–0.8 mg/mL. When the concentration was increased from 0.8–1.6 mg/mL, the change of the scavenging rate was not obvious any longer, indicating the derivatives’ optimum concentration in scavenging hydroxyl radicals was at around 0.8 mg/mL. For native starch, the obvious decrease of the scavenging rate could be observed at 0.2 mg/mL. Considering the three times repeat of the test, this phenomenon could be reasoned by the low water solubility and insufficient antioxidant activity of the native starch. The enhanced water solubility of derivatives due to the introduction of the hydrophilic 1,2,3-triazole and positive charges, partially broke the initial hydrogen bonds, resulting the hydroxyl of derivative more exposed to the outside through a direct route on the triazole ring. As a result, these hydroxyls could react with free radicals to form most stable compounds by typical H-abstraction reaction or through addition reaction. Moreover, the scavenging abilities of 2a–2e were stronger than that of 1a-1e after the alkylation with methyl iodide. This was possible due to the added positive charges as a stabilizer for free radicals.

DPPH radical is a very stable nitrogen-centered free radical due to the conjugation and steric hindrance effect, which is considered as one of the standard and easy colorimetric methods for the evaluation of antioxidant properties of polysaccharide [37]. Under a mild alkaline condition, NADH can be oxidized by PMS to generate superoxide anion free radical, which can further react with NBT to form a blue substance, namely formazan. The maximum UV absorption wavelength of the formazan solution is located at 560 nm. When an efficient sample is added, it can be preferentially combined with the superoxide anion free radical, thus preventing the generation of the colored substance. In this way, the sample with lighter color has a stronger superoxide scavenging ability.

**Figure 4:**
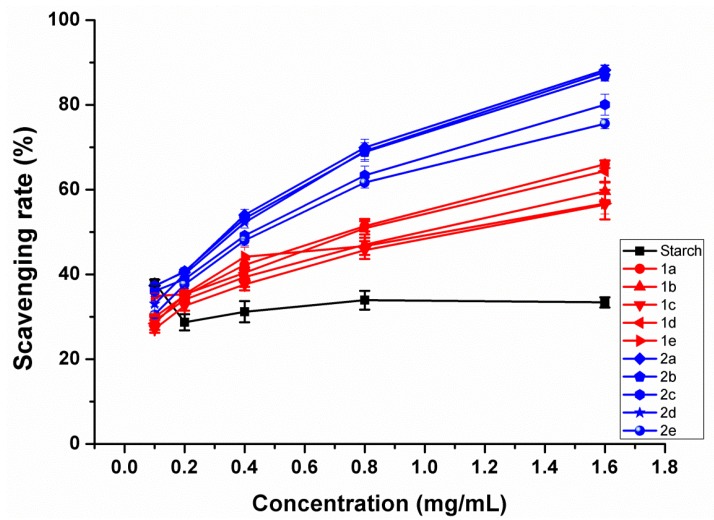
DPPH radical scavenging activity of starch and starch derivatives.

The scavenging abilities of starch and its derivatives against DPPH radicals are shown in Figure 4. The results were similar to the hydroxyls radical scavenging activity caused by derivatives. These derivatives also exhibited distinct antioxidant activity compared to starch. After the cationization of 1,2,3-triazole, derivatives of 2a–2e had an improved antioxidant activity. R Song et al. [38] reported that the hydroxyl groups had an important effect in scavenging DPPH radicals. DPPH radicals could be trapped by antioxidants through the hydrogen donation, forming a stable DPPH-H molecule.

Superoxide free radical is a kind of free radical produced in the metabolic process of organisms. It can directly attack biological macromolecules and additionally act as a precursor of hydroxyl radical and H_2_O_2_, causing damage to cell structures and functions [39]. Superoxide may be considered the ultimate danger for the aerobic life, because it is unavoidably produced and is potentially toxic [40].

**Figure 5:**
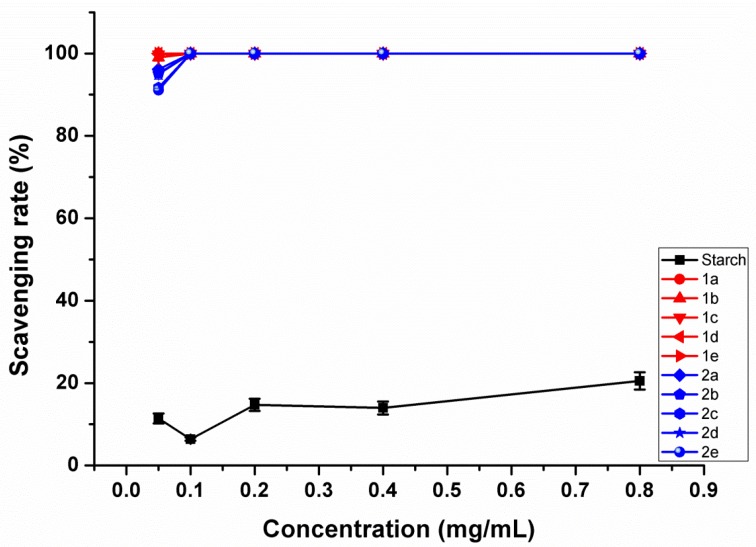
Superoxide radical scavenging activity of starch and starch derivatives.

The superoxide radical scavenging abilities of the synthesized starch derivatives are shown in Figure 5. A very strong scavenging effect of the derivatives against superoxide radicals can be observed in the figure. Even though the test concentration dropped to 0.05 mg/mL, the scavenging rate was still close to 100% and far beyond that of starch. Unlike the results from the DPPH and hydroxyl radicals scavenging assays, derivatives of 1a–1e had a stronger scavenging ability than 2a–2e against superoxide radicals at 0.05 mg/mL. There were no related reports to explain this phenomenon. So, further research is needed in the future. But when the concentration beyond 0.05 mg mL^−1^, all the derivatives including 1a–1e and 2a–2e could completely scavenge the superoxide radicals.

### 3.5. Hemolysis Assay

**Figure 6:**
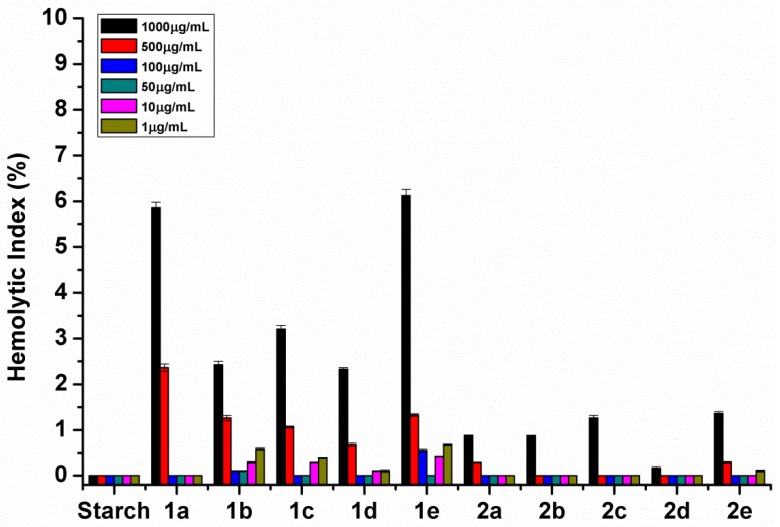
Hemolysis effect of starch and starch derivatives.

The synthesized cationic starch derivatives (2a–2e) with pronounced antioxidant activity compared to native starch have great potential when utilized in the food and biomedical fields. As a result, the hemolysis assay of the derivatives as a preliminary assessment of the biocompatibility is necessary. The results of the hemolysis assay are shown in Figure 6. The hemolysis rate of native starch was 0% and it was irrelevant to the concentration. The result was consistent with its favorable biocompatibility. Except for 1a and 1e, the hemolysis rates of all the test samples were lower than 5%. This was an acceptable value for biomaterial applications [34]. Regarding 1a and 1e, their hemolysis rates were slightly higher than 5% only when the concentration arrived at 1000 μg/mL. Moreover, the hemolysis rates of all the cationic starch derivatives (2a–2e) at the test concentrations were lower than 2%. According to ASTM F756-17 [41], biomedical materials with hemolysis rates under 2% were considered to be non-hemolytic. As a result, the cationic starch derivatives (2a–2e) had more advantages than the precursor starch derivatives (1a–1e) in terms of hemolysis effects. The results further confirmed the prospect of the starch derivatives utilized in the food and biomedical fields.

## 4. Conclusions

In summary, five cationic 1,2,3-triazole functionalized starch derivatives were synthesized by using the “click” reaction and *N*-alkylation. A convenient method for pre-azidation of starch was developed. The structures of the obtained derivatives were analyzed using FTIR and ^1^H NMR. Their antioxidant activities were evaluated by testing their radicals scavenging activities against hydroxyl radicals, DPPH radicals and superoxide radicals. All the starch derivatives including 1a–1c and 2a–2c exhibited pronounced antioxidant activity compared to pure starch and they are particularly effective against superoxide radicals. These results demonstrated the advantages of chemical modification in improving the antioxidant activity of starch. The improved antioxidant activities of the derivatives probably benefited from the enhanced water solubility and the added positive charge. The biocompatibility of the cationic derivatives was confirmed by the low hemolytic rate (<2%). The starch derivatives described in this study have great potential as antioxidant materials for food and biomedical applications.

## Figures and Tables

**Table 1:** The yield, water solubility, and the degree of substitution of starch derivatives.

Compound	Yield	Water Solubility (mg/mL)	Degree of Substitution
Starch	/	/	/
CASC	94%	207 ± 3.8	1.04
AASC	58%	322 ± 2.4	0.42
1a	54%	522 ± 1.1	0.24
1b	48%	515 ± 1.7	0.28
1c	47%	507 ± 1.9	0.25
1d	45%	486 ± 1.7	0.24
1e	50%	492 ± 1.4	0.22
2a	64%	835 ± 2.1	0.22
2b	57%	844 ± 2.5	0.21
2c	59%	840 ± 2.2	0.22
2d	54%	851 ± 1.8	0.24
2e	42%	825 ± 2.4	0.20
